# A position statement and practical guide to the use of particulate filtering facepiece respirators (N95, FFP2, or equivalent) for South African health workers exposed to respiratory pathogens including *Mycobacterium tuberculosis* and SARS-CoV-2

**DOI:** 10.7196/AJTCCM.2021.v27i4.173

**Published:** 2021-10-22

**Authors:** K Dheda, S Charalambous, A S Karat, A von Delft, U G Lalloo, R van Zyl Smit, R Perumal, B W Allwood, A Esmail, M L Wong, A G Duse, G Richards, C Feldman, M Mer, K Nyamande, U Lalla, C F N Koegelenberg, F Venter, H Dawood, S Adams, N A B Ntusi, H-M van der Westhuizen, M-Y S Moosa, N A Martinson, H Moultrie, J Nel, H Hausler, W Preiser, L Lasersohn, H J Zar, G J Churchyard

**Affiliations:** 1 Centre for Lung Infection and Immunity, Division of Pulmonology, Department of Medicine and UCT Lung Institute and South African MRC/UCT Centre for the Study of Antimicrobial Resistance, University of Cape Town, Cape Town, South Africa; 2 Faculty of Infectious and Tropical Diseases, Department of Immunology and Infection, London School of Hygiene and Tropical Medicine, London, United Kingdom; 3 The Aurum Institute, Johannesburg, South Africa; 4 School of Public Health, University of the Witwatersrand, Johannesburg, South Africa; 5 TB Centre, London School of Hygiene and Tropical Medicine, London, United Kingdom; 6 School of Public Health and Family Medicine, University of Cape Town, Cape Town, South Africa; 7 TB Proof, South Africa; 8 Gateway Private Hospital Medical Centre, Umhlanga Ridge, South Africa; 9 Durban International Clinical Research Site, Durban, South Africa; 10 Division of Pulmonology and Department of Medicine, University of Cape Town and Groote Schuur Hospital, Cape Town, South Africa; 11 Division of Pulmonology, Department of Medicine, Stellenbosch University and Tygerberg Hospital, Cape Town, South Africa; 12 Clinical Trials Unit, University of Cape Town Lung Institute, South Africa; 13 Division of Pulmonology, Department of Medicine, School of Clinical Medicine, Faculty of Health Sciences, University of the Witwatersrand, Johannesburg, South Africa; 14 Clinical Microbiology & Infectious Diseases, School of Pathology of the NHLS & University of the Witwatersrand, Johannesburg, South Africa; 15 Department of Critical Care, Faculty of Health Sciences, University of the Witwatersrand, Johannesburg, South Africa; 16 Department of Internal Medicine, School of Clinical Medicine, Faculty of Health Sciences, University of the Witwatersrand, Johannesburg, South Africa; 17 Department of Medicine, Divisions of Pulmonology and Critical Care, Charlotte Maxeke Johannesburg Academic Hospital and Faculty of Health Sciences, University of the Witwatersrand, Johannesburg, South Africa; 18 Department of Pulmonology, Nelson R Mandela School of Medicine, College of Health Sciences, University of KwaZulu Natal, Durban, South Africa; 19 Ezintsha, Faculty of Health Sciences, University of the Witwatersrand, Johannesburg, South Africa; 20 Greys Hospital, Pietermaritzburg, South Africa; 21 Division of Occupational Medicine, School of Public Health and Family Medicine, University of Cape Town, South Africa; 22 Division of Cardiology, Department of Medicine, University of Cape Town and Groote Schuur Hospital, Cape Town, South Africa; 23 Nuffield Department of Primary Care Health Sciences, University of Oxford, United Kingdom; 24 Department of Infectious Diseases, Division of Internal Medicine, Nelson R Mandela School of Medicine, University of KwaZulu-Natal, Durban, South Africa; 25 Southern African HIV Clinicians Society; 26 Perinatal HIV Research Unit (PHRU), University of the Witwatersrand, Johannesburg, South Africa; 27 Johns Hopkins University Center for TB Research, Baltimore, MD, USA; 28 National Institute for Communicable Diseases, Division of the National Health Laboratory Service, Johannesburg, South Africa; 29 Clinical Microbiology & Infectious Diseases, School of Pathology, Faculty of Health Sciences, University of the Witwatersrand, Johannesburg, South Africa; 30 Division of Infectious Diseases, Department of Medicine, University of the Witwatersrand, Johannesburg, South Africa; 31 TB HIV Care, Cape Town, South Africa; 32 Division of Medical Virology, Faculty of Medicine and Health Sciences, Stellenbosch University and National Health Laboratory Service Tygerberg, Cape Town, South Africa; 33 South African Society of Anaesthesiologists; 34 Department of Anaesthesia, Faculty of Health Sciences, University of the Witwatersrand, Johannesburg, South Africa; 35 Division of Critical Care, Chris Hani Baragwanath Hospital and University of the Witwatersrand, Johannesburg, South Africa; 36 Department of Paediatrics & Child Health, Red Cross Children’s Hospital and SAMRC Unit on Child and Adolescent Health, University of Cape Town, South Africa; 37 Department of Medicine, Vanderbilt University, Nashville, Tennessee, USA

## Abstract

**Summary:**

Severe acute respiratory syndrome coronavirus-2 (SARS-CoV-2) is transmitted mainly by aerosol in particles <10 µm that can remain suspended for hours before being inhaled. Because particulate filtering facepiece respirators (‘respirators’; e.g. N95 masks) are more effective than surgical masks against bio-aerosols, many international organisations now recommend that health workers (HWs) wear a respirator when caring for individuals who may have COVID-19. In South Africa (SA), however, surgical masks are still recommended for the routine care of individuals with possible or confirmed COVID-19, with respirators reserved for so-called aerosol-generating procedures. In contrast, SA guidelines do recommend respirators for routine care of individuals with possible or confirmed tuberculosis (TB), which is also transmitted via aerosol. In health facilities in SA, distinguishing between TB and COVID-19 is challenging without examination and investigation, both of which may expose HWs to potentially infectious individuals. Symptom-based triage has limited utility in defining risk. Indeed, significant proportions of individuals with COVID-19 and/or pulmonary TB may not have symptoms and/or test negative. The prevalence of undiagnosed respiratory disease is therefore likely significant in many general clinical areas (e.g. waiting areas). Moreover, a proportion of HWs are HIV-positive and are at increased risk of severe COVID-19 and death.

**Recommendations:**

Sustained improvements in infection prevention and control (IPC) require reorganisation of systems to prioritise HW and patient safety. While this will take time, it is unacceptable to leave HWs exposed until such changes are made. We propose that the SA health system adopts a target of ‘zero harm’, aiming to eliminate transmission of respiratory pathogens to all individuals in every healthcare setting.

Accordingly, we recommend:

1. the use of respirators by all staff (clinical and non-clinical) during activities that involve contact or sharing air in indoor spaces with individuals who: (*i*) have not yet been clinically evaluated; or (*ii*) are thought or known to have TB and/or COVID-19 or other potentially harmful respiratory infections;
2. the use of respirators that meet national and international manufacturing standards;
3. evaluation of all respirators, at the least, by qualitative fit testing; and
4. the use of respirators as part of a ‘package of care’ in line with international IPC recommendations.

We recognise that this will be challenging, not least due to global and national shortages of personal protective equipment (PPE). SA national policy around respiratory protective equipment enables a robust framework for manufacture and quality control and has been supported by local manufacturers and the Department of Trade, Industry and Competition. Respirator manufacturers should explore adaptations to improve comfort and reduce barriers to communication. Structural changes are needed urgently to improve the safety of health facilities: persistent advocacy and research around potential systems change remain essential.

## Background

Almost 2.5 million South Africans have tested positive for severe acute respiratory syndrome coronavirus-2 (SARS-CoV-2) since March 2020, and >90 000 have died in hospitals from COVID-19.^[1]^ Although SARS-CoV-2 was initially thought to spread predominantly through droplet or direct contact, there is strong evidence that aerosol-based transmission is likely the dominant route of spread.^[2]^ This is especially important in the light of the circulation (at the time of writing) of the Delta variant, which is more transmissible than the original virus or Beta variant.^[3]^ Clinically distinguishing people with COVID-19 from those with other respiratory infections is impossible without testing. This is because many people infected with SARS-CoV-2 are asymptomatic (estimates of asymptomatic proportions vary widely from <20 to >90%),^[4,5]^ and because many respiratory symptoms experienced are often nonspecific.^[6]^ Frontline health workers (HWs) are highly exposed and at high risk of infection, as shown by the thousands who have been infected, developed illness, and died.^[7]^

HWs in high tuberculosis (TB) burden countries are already at high risk of TB infection and disease.^[8–11]^ Despite significant progress, TB incidence in South Africa (SA) remains high at over 600 per 100 000 population (around 360 000 new cases per year), and it has consistently been the country’s leading cause of death, responsible for ~60 000 deaths every year.^[12]^ Management of a respiratory pandemic is more complex in high TB burden countries such as SA. In addition to previous or current TB, individuals seeking care often have a history of one or more of HIV, tobacco smoking, biomass fuel exposure, outdoor air pollution, or exposure to mine dust containing silica, which considerably expands the differential diagnosis in those presenting with respiratory symptoms.^[13,16]^ Presentations and risk factors can be difficult to differentiate without additional time and investigation, both of which can increase the likelihood and duration of HW exposure to infectious individuals. Current or previous TB may also place people at increased risk of developing COVID-19,^[17]^ and having COVID-19 may increase the risk of TB, though reliable data are not yet available.

International guidelines recommend that HWs should be wearing N95 or equivalent particulate filtering facepiece respirators (‘respirators’) for routine care of individuals with possible TB or COVID-19, although World Health Organization [WHO] COVID-19 guidelines are not entirely consistent.^[18,19]^ At the time of writing, however, SA COVID-19 guidelines state that this level of protection is needed only for ‘aerosol-generating’ procedures (AGPs), such as intubation and bronchoscopy.^[19–21]^ Recent studies suggest that coughing – common in both TB and COVID-19 pneumonitis – may produce as much (or more) aerosol than some AGPs.^[22–24]^ There is also strong evidence that, like *Mycobacterium tuberculosis* (*Mtb*),^[2,25]^ SARS-CoV-2 is also transmitted by aerosol. The data to support this are wide-ranging, and include outbreak investigations,^[2]^ experiments showing virus viability in aerosols for up to 3 hours,^[26]^ detection of viable virus in air samples from COVID-19 infected persons and animals,^[27,29]^ and identification of SARS-CoV-2 in air filters and ducts.^[30]^ In other studies, activities like speaking, shouting, and singing have been shown to produce substantial amounts of infectious aerosol, and the use of high-flow oxygen may also increase aerosol propagation.^[31,32]^ The definition of an AGP in SA guidelines is therefore overly restrictive^[21]^ and there is a pressing need to ensure that HWs are adequately protected from both *Mtb* and SARS-CoV-2.

In this position statement, we build the case for national policies to support more widespread and consistent use of respirators by HWs in high-TB-burden countries such as SA, both during the ongoing COVID-19 pandemic and beyond. We make specific recommendations for situations in which respirators should be worn by HWs in SA and discuss some of the additional efforts needed to sustain this policy.

## Making the argument

### Health workers are left unprotected

Effective implementation of infection prevention and control (IPC) in healthcare facilities is important to avoid ‘institutional amplification’ of epidemics,^[33,35]^ to protect the people who attend and work there, and to preserve the health workforce – a critical issue at all times, and more so during a pandemic. TB in HWs is a persistent problem: numerous studies over 30 years have shown that HWs in high-TB-burden countries are more likely than the general population to develop both latent TB and active TB disease, largely because of occupational exposure.^[8,9,36-39]^ Many HWs may have underlying vulnerability to severe disease and require extra protection.^[40,41]^ In addition to the risk to HWs themselves, there are also risks of transmission to their families, vulnerable household members, and patients. The thousands of HWs who have developed and died from COVID-19 in the last 18 months clearly demonstrate both the risks faced by HWs and the insufficient priority given to HW safety.^[7,42-45]^

HWs, like all other SA workers, have a right to a healthy and safe working environment.^[46]^ HWs worldwide have paid a disproportionate price for governments’ and health systems’ lack of foresight, lack of preparation, and underinvestment in pandemic preparedness.^[47]^ This has manifested, among other things, as inadequate or insufficient personal protective equipment (PPE) for frontline and other staff.^[48,49]^ Indeed, it was shown recently that none of the tested ‘KN95’ respirators evaluated in SA met stipulated safety standards for HW protection.^[50]^ We echo calls by other authors for urgent research, funding, and prioritisation of IPC and HW protection^[51]^ and for more comprehensive approaches to occupational health.^[52,53]^ Though we recognise that systemic changes will take time to enact, it is unacceptable that HWs remain at risk until such changes are made.

### On the frontline, it is impossible to differentiate between TB, SARS-CoV2 and other infections

It is near impossible to make a specific diagnosis of TB, COVID-19, or other respiratory disease in most SA healthcare facilities without detailed clinical assessment and laboratory investigation. This usually takes at least 24 - 72 hours (the turnaround time of most diagnostic tests). Clinical diagnosis is difficult because there are overlapping risk factors, a multitude of possible presentations (including non-specific symptoms such as fever or cough), and often more than one infection. ^[13-15,17,54,55]^ If assessing HWs are not adequately protected, this can be a major opportunity for transmission to occur. Transmission of SARS-CoV-2 by asymptomatic individuals has been widely documented,^[6]^ and the burden of ‘subclinical’ TB^[56]^ in SA is increasingly evident. The recent national TB prevalence survey found that around half of the people in the community with confirmed pulmonary TB did not have symptoms suggestive of TB^[57]^ and a recent study in KwaZulu-Natal showed similar findings among adults attending primary healthcare (PHC) clinics.^[58]^

Symptom screening cannot differentiate between TB and other respiratory infections. TB can often present as acute pneumonia or acute lower respiratory tract infection (LRTI), and *Mtb* may be among the most common pathogens isolated in this context in Asian and African settings (reviewed in detail in a recent article).^[54]^ In a large study from SA (*n*=2 500 patients), a symptom duration threshold of >14 days was unable to distinguish between TB and other respiratory pathogens, and in those with LRTI of <14 days duration, TB was the microbiologically-proven diagnosis in ~18% of patients.^[59]^ This figure is remarkable, considering that in patients with acute LRTI, a microbiological diagnosis is made in only ~50% of cases.

Triage based on symptom screening is challenging to perform consistently and has been shown to be sub-optimally implemented at PHC clinics across the country.^[60,61]^ In addition, adults accompanying children or other vulnerable individuals may not be screened and may be undetected sources of *Mtb* or SARS-CoV-2. This means that potentially infectious individuals with undetected disease (with or without symptoms) may remain in general patient streams in perceived ‘lower-risk’ areas, with subsequent inappropriate use of less effective PPE by HWs.

As discussed below, current guidance recommends use of different PPE for different ‘types’ of patients (e.g. respirators only when in contact with individuals with ‘possible’ or ‘known’ TB or COVID-19). As we have emphasised, however, it is almost impossible to estimate who is likely to have TB or COVID-19 (or another respiratory infection, such as influenza or bacterial or fungal pneumonia). It is also difficult to reliably estimate the risk of transmission in any given space at any given time without information on infectiousness, ventilation, occupancy of rooms, and duration of exposure.^[62]^ It is therefore unrealistic to expect individual HWs to make repeated assessments of risk during the course of a working day and adjust their PPE accordingly, particularly at a time when the health system is under pressure.^[63,64]^

### SARS-CoV-2 and *Mtb* are transmitted by aerosol: particulate filtering facepiece respirators offer better protection

Person-to-person transmission of SARS-CoV-2 is currently understood to occur predominantly by two routes. First, via larger respiratory droplets (>10 µm), which fall rapidly to the ground or onto surfaces – droplets of size 10 µm and 100 µm take ~10 minutes and ~6 seconds, respectively, to fall to the ground.^[65]^ Such droplets may be inhaled or deposited into the nasopharynx or directly inoculated onto mucous membranes (eyes, mouth, upper pharynx) or the skin, with subsequent person-to-person transfer via direct contact or infected fomites. Second, via aerosol: particles produced through coughing, speech, singing, and AGPs, which after desiccation are usually up to 10 µm in diameter, can remain suspended in the air for several hours, and may be inhaled into the lungs (small airways and alveoli) of an exposed person.^[25,66-69]^ The second route, sometimes referred to as airborne transmission, is also the main route of transmission of the measles virus and *Mtb*. ^[62,70]^ However, is important to note that terminology (airborne v. aerosol v. respiratory droplets) is not standardised or well defined, and thus airborne spread is likely to be due to production of a continuum of virus-containing droplet sizes that may be deposited in the upper and/or lower respiratory tract of susceptible individuals.^[25]^

Early in the pandemic, SARS-CoV-2 was thought to be transmissible only via large droplets/fomites, and precautions therefore centred on restricting close contact, cleaning surfaces, and handwashing.^[71,72]^ However, research on aerosolisation has shown that respiratory particle sizes vary widely, and that smaller particles (<5 µm) are more likely to contain pathogen.^[25]^ The high likelihood of aerosol transmission of SARS-CoV-2^[2,73-75]^ has now been acknowledged by WHO and the United States Centers for Disease Control and Prevention (US CDC), which recently made recommendations around improving ventilation of indoor spaces as part of coronavirus-related IPC.^[76,77]^ Ventilation is important for mitigating aerosol transmission: increasing the number of air changes per hour means that suspended particles are more likely to be removed before they can be inhaled.^[78,79]^ Ten cogent reasons including underlying evidence as to why aerosol-based transmission is an important and co-dominant route of SARS-CoV-2 transmission were recently elegantly summarised in the *Lancet*. ^[2]^

The evidence around the relative efficacy of masks and respirators against aerosols is also reasonably clear. In laboratory studies, respirators (filtering facepiece (FFP) 2 or FFP3) were shown to be 16 - 108 times more effective than fluid-repellent surgical masks (FRSMs) in filtering aerosolised sodium chloride.^[80]^ Clinical studies are less definitive, in part because of variation in methodologies and definitions of exposure, and issues with the power of the studies. At least two studies, however, have shown statistically important reductions in risk with use of quality respirators compared with surgical masks, particularly when used continuously (as opposed to ‘targeted’ use) and when individuals were exposed to clinical respiratory illness.^[80–84]^

### Current South African guidance is at odds with international recommendations

Table 1 summarises SA and global guidance around respirator use for personal protection against *Mtb* and SARS-CoV-2. The majority of national and international bodies (other than Public Health England)^[85]^ recommend the use of respirators for routine care of individuals with possible or confirmed COVID-19 or TB. SA, at present, recommends an N95 respirator for care of individuals with possible or confirmed TB (in line with 2001 legislation around hazardous biological agents),^[86]^ but only a surgical mask for routine care of individuals with possible or confirmed COVID-19, which is at odds with recent employment legislation.^[21,87-89]^ This does not offer individuals protection against aerosol transmission of SARS-CoV-2. Importantly, this also requires HWs to differentiate between those who may have TB and those who may have COVID-19, which, as outlined above, may be impossible to do without risking exposure.

In summary (and sidestepping controversies about whether surgical masks or respirators are essential for protection against SARS-CoV-2), the high risk of HW exposure to and infection with *Mtb* and inability to differentiate TB from other acute LRTIs mandates the consistent use of respirators by HWs in high TB burden settings. It is impractical and not clinically meaningful to provide pathogen-specific guidance on masking. We have therefore provided guidance below in the context of routine exposure to acute respiratory infections.

**Table 1:** South African and international recommendations for use of masks and respirators by health workers (terms used are consistent with those in the respective guidelines)*

		**TB**		**COVID-19**	
**Source**	**Scope**	**Routine care^#^**	**AGPs^†^**	**Routine care^#^**	**AGPs^†^**
**SA DoH/NICD^[21,87]^**	SA	N95 respirator	N95 respirator	**Surgical mask**	N95 respirator
**WHO^[19,20]^**	Global	Particulate respirator (high- TB-burden settings)^‡^	Particulate respirator	N95/FFP2/FFP3 respirator^§^	N95/FFP2/FFP3 respirator
**US CDC^[90,91]^**	USA	N95 respirator (at least)	N95 respirator at least. Consider elastomeric full-facepiece respirator or PAPR	N95 or equivalent or higher-level respirator	N95 respirator or respirators that offer a higher level of protection
**PHE^[85]^/NICE^[92]^**	England/ United Kingdom	FFP2 respirator	FFP2 or FFP3 respirator	**Fluid-resistant surgical face mask (Type IIR)**	FFP3 respirator or hood
**ECDC^[93,94]^**	Europe	Respirator	Respirator	Respirator	Respirator
**India MoHFW^[95,96]^**	India	N95 respirator	N95 respirator	N95 respirator^§^	N95 respirator

TB = tuberculosis; COVID-19 = coronavirus disease 2019; AGP = aerosol-generating procedure;

DoH = Department of Health; NICD = National Institute for Communicable Diseases; US CDC = United States Centers for Disease Control and Prevention

WHO = World Health Organization; ECDC = European Centre for Disease Control and Prevention; FFP = filtering facepiece

IDSA = Infectious Diseases Society of America; n/s = not specified; NICE = National Institute for Health and Care Excellence

PAPR = powered air-purifying respirator; PHE = Public Health England; MoHFW = Ministry of Health and Family Welfare

* Terms used for mask/respirator types are consistent with those used in the respective guidelines. WHO defines ‘particulate respirators’ as those meeting N95 or FFP2 standards. ECDC defines ‘respirators’ as those meeting FFP2 or FFP3 standards.

# Routine care of people with possible or confirmed TB or COVID-19.

† AGPs (aerosol-generating procedures) include the following: endotracheal intubation/extubation; respiratory tract suctioning; manual ventilation; tracheotomy; tracheostomy; bronchoscopy; surgery or post mortems involving high-speed cutting (of the respiratory tract); certain dental procedures; non-invasive and high-frequency oscillating ventilation; use of high-flow nasal oxygen, sputum induction; chest physiotherapy; cardiopulmonary resuscitation; and collection of naso- and oropharyngeal swabs.

‡ ‘to reduce M. tuberculosis transmission to health workers, persons attending healthcare facilities or other persons in settings with a high risk of transmission’

§ ‘…for work with infected people in indoor, crowded places without adequate ventilation’.

§ ‘…in all patient care areas, while providing patient care’.

## Recommendations

### Zero transmission, zero harm: Our recommendations for the widespread consistent use of particulate respirators in high TB burden settings

We propose that the health system should aim for a target of ‘zero transmission, zero harm’: a position that builds on the precautionary principle^[97]^ and the foundational ethical value of ‘do no harm’ to suggest that the health system’s duty of care extends beyond patients to include its workforce. The principle of ‘zero harm’ has been used most widely to refer to efforts to improve patient safety,^[98]^ but here we use the term specifically around disease transmission. It is unacceptable that any person should be infected with *Mtb* or SARS-CoV-2 because of exposure in a healthcare facility, and the health system should aim to eliminate transmission in all healthcare settings. Clearly, this will require prioritisation and significant long-term investment in a range of IPC measures. These include consideration of building design, ventilation, ultraviolet germicidal irradiation (UVGI) systems, and organisation of services to reduce overcrowding and enable consistent implementation of administrative measures (such as triage, respiratory isolation, prompt treatment, and disinfection of surfaces and equipment). This also means that HWs are entitled to, and should have access to, high-quality PPE sufficient to protect against both droplet and aerosol transmission, with efforts made to minimise exposure.

We therefore make the following recommendations:

1. Particulate FFP respirators should be worn by:

- all staff (clinical and non-clinical) during activities that involve contact or sharing air in indoor spaces (more so if poorly ventilated) with individuals who (*i*) have not yet been clinically evaluated or (*ii*) are thought or known to have TB and/or COVID-19 (this will likely include waiting areas, emergency departments, clinic consultation rooms, and certain inpatient wards and high care/intensive care units);
- frontline staff in clinical areas who are in contact with patients thought or known to have TB, COVID-19, or other respiratory infection, including influenza, measles, and varicella (likely areas include emergency departments, medical admissions units, and ‘patients under investigation’ (PUI) wards); and
- any staff involved in high-risk or aerosolising procedures involving individuals thought or known to have TB or COVID-19 (e.g. bronchoscopy, open or closed suctioning of the airway, non-invasive ventilation, oxygen, and dental procedures, among others).

2. Respirators (N95, FFP2, and other equivalent respirators, e.g. quality-assured KN95 masks) should fulfil the following requirements, per criteria set out by SA National Department of Health (NDoH)^[99]^ and the USA National Institute for Occupational Safety and Health (NIOSH; see Box 1).^[100]^

Box 1 **Quality requirements for particulate filtering facepiece respirators in South Africa.**

**Certification**

All particulate filtering facepiece respirators sold should be accompanied by Homologation Certificates, proof of international compliance (in the case of imported RPE including NIOSH approvals, European Union certifications, CE marking reports, and complete FDA registrations) and quality certificates. The minimum required stipulation is:

1. Total inward leakage using quantitative and/or qualitative fit tests (performed at facility level on individuals);
2. Determination of particulate filter penetration (PFP) with the minimum testing requirement being to NaCl filtration (and only where possible to paraffin oil and latex particles);
3. Determination of flow resistance (inhalation resistance at a minimum, but preferably inhalation and exhalation resistance with the latter mandatory for valved respirators);
4. Flammability testing;
5. Fluid resistance test (this test is not mandatory at this time owing to capacity and development constraints in lab testing in SA). Where fluid resistance testing has not been conducted by a verified international lab but all other local tests pass, the recommendation is for mandatory visor usage to protect against respirator fluid exposure.

**Metrology notification**

All filtering facepiece respirators (SAHPRA Class B device) in the interests of identification, safety and to ensure that homologation is possible and accurate should have a clear physical marking/stamp on each mask or respirator with the mandatory (in bold) minimum information being:

1. **Manufacturer/brand name/registered trademark** or easily understood abbreviation,
2. **The mask or respirator efficiency classification**; an alphanumeric rating as recognised (e.g., FFP2, FFP3, N95, KN95);
3. **Standard compliance label** that indicates the local SANS standard showing the device has been tested against and passed;
4. **Size of the respirator, model number and lot number**; and
5. **Any other mandatory markings** as required by SANAS, NRCS, SAHPRA, other national regulator or standard and as may be required by the Legal Metrology Act, 2014 (Act 9 of 2014).

FDA = United States Food and Drug AdministrationNRCS = National Regulator for Compulsory SpecificationsRPE = respiratory protective equipmentSAHPRA = South African Health Products Regulatory AuthoritySANAS = South African National Accreditation SystemSANS = South African National StandardAdapted from the SA National Department of Health’s Policy for the Regulation of Quality Respiratory Protective Equipment (RPE) Supply in Healthcare (2020).^[99]^

- All respirators should be accompanied by Homologation Certificates, proof of international compliance, and quality certificates. The filter designation, manufacturer, model number, and certification approval number should be displayed on the body of the respirator.
- All respirators require a clear physical marking with (*i*) the manufacturer/brand name/registered trademark; (*ii*) an alphanumeric rating as recognised (e.g. FFP2, FFP3, N95, KN95); (*iii*) a standard compliance label showing the standard/s the device has met; (*iv*) the size of the respirator, model number, and lot number; and (*v*) any other mandatory markings.

The respirator should, at minimum, be evaluated by qualitative fit testing.^[101]^ Fit testing forms an indispensable part of achieving the objective filtration of virus and bacteria and should be carried out at least annually for every HW required to wear a respirator, in accordance with the respirator’s brand and size. Additional fit testing is generally recommended if the subject experiences a weight change of ≥10 kg or has significant dental changes, reconstructive surgery, or facial disfigurement.^[99]^ We recommend that healthcare facilities have access to low-cost qualitative fit testing equipment (e.g. the 3M Qualitative Fit Test Apparatus FT-10 (3M, SA))^[50]^ so that respirators and wearers can be evaluated. Qualitative fit testing is simpler and cheaper than quantitative testing. This may be at individual healthcare facilities or through local or regional centres. This will also provide regulatory bodies an opportunity to evaluate masks that claim to meet N95 or FFP2 standards and will go towards establishing a ‘respiratory protection programme’ for HWs, in line with international guidance.^[20]^ To meet new recommendations by the South African Health Products Regulatory Authority (SAHPRA),^[102]^ regional or national comprehensive testing nodes should be established to perform more rigorous quantitative fit testing (for example, using the ambient aerosol condensation nuclei counter protocol)^[101]^ and evaluation of filtration integrity. There is currently negligible access to such facilities in the SA public or private sectors.

3. It is critical to emphasise that particulate respirators alone are less likely to be effective if other IPC measures are not implemented. Therefore, in line with international IPC guidelines,^[19,20]^ a ‘package of care’ approach should be adopted, the major elements of which are detailed in Table 2.

**Table 2:** Major elements of a ‘package of control’ approach to infection prevention and control for airborne infections in healthcare facilities

**Category**	**Details or examples**
**Administrative**	Examples include triage and separation of people with infectious or potentially infectious TB, COVID-19, and/or influenza, etc.				
**Environmental**	E.g., ensuring good ventilation (minimum 6 - 12 air changes per hour equivalent), minimising crowding, and using UVGI.				
**Personal protection**	Using high quality PPE as appropriate (e.g., respirators, eye protection, gloves, aprons).				
**Additional measures to reduce transmission between clinic attendees and from clinic attendees to HWs.**	E.g., face coverings for all individuals attending health facilities (source control), physical distancing, and hand hygiene				
**Additional measures to reduce transmission between HWs.**	Attention to IPC in non-clinical areas such as staff canteens, rest areas, and changing rooms				
**Additional measures to reduce transmission to and from HWs outside of healthcare facilities.**	HWs trained to maintain precautions outside of health facilities. For example, during use of public transport; by minimising time spent in poorly ventilated, densely occupied areas; and by maintaining physical distancing, hand hygiene, and use of face coverings.				
**Longer term measures to strengthen systems and reduce risks of transmission.**	Respiratory protection programmes; surveillance for healthcare-associated infections; monitoring/audit of IPC practices with feedback*				

TB = tuberculosis

COVID-19 = coronavirus disease 2019

UVGI = ultraviolet germicidal irradiation

HW = health worker

PPE = personal protective equipment

IPC = infection prevention and control

* See ‘core components of IPC programmes’ in 2019 WHO TB IPC guidelines.^[20]^

We also note that the practice of wearing a surgical mask over a fit tested quality respirator to prevent contamination or improve efficacy is not evidence based, has not been evaluated scientifically, and may unnecessarily increase the work of breathing. Wearing a surgical mask underneath a respirator is not recommended as it is likely to compromise fit and therefore the efficacy of the respirator. In addition, given the occurrence of breakthrough infections with SARS-CoV-2 in individuals who have received a partial or even full vaccination course,^[103]^ no differentiation should be made according to vaccination status as regards to use of PPE.

## Hurdles and challenges

Our aim is to make recommendations for measures that will provide the highest level of protection to HWs and patients, regardless of the logistical obstacles. We recognise, however, that these recommendations may not be straightforward to implement. Health systems have been severely affected by the global shortage of quality respirators and are facing challenges with procurement and manufacturing. The NDoH should work with manufacturers, regulatory bodies, and other relevant parties to find ways to overcome challenges to better serve HWs. We also urge manufacturers to explore improving the comfort of respirators and to take measures to reduce barriers to communication (e.g. by using transparent materials to allow lipreading). Innovative methods should be explored to produce new masks (e.g. 3D printing) without compromising on quality.^[104,105]^

The US CDC states that respirators are ‘meant to be disposed after each use’, but also describes contingency strategies in the case of acute shortages or crisis, including ‘decontamination’ (e.g. with UVGI, hydrogen peroxide, or moist heat, also known as ‘reprocessing’), ‘extended use’ (continuous use of the same respirator for encounters with multiple patients), and ‘limited reuse’ (use of the same respirator for encounters with multiple patients, with the respirator donned and doffed between encounters).^[106]^ Each approach carries risks, most importantly of reductions in respirator fit and filtration performance, but also of contamination and self-contamination through repeated donning and doffing.^[107-109]^ As such, SAHPRA and NDoH currently prohibit decontamination/reprocessing of respirators by any method but, in the case of shortages or if supply optimisation is required, do support extended use (with no attempts at cleaning or decontaminating and ideally without repeated donning and doffing) of single-use respirators for up to 6 - 8 hours, depending on the manufacturer.^[99]^

From a long-term IPC perspective, a focus on respirator use risks over-emphasising individual protection, shifting responsibility back to individual HWs and lessening pressure on the healthcare system to make the structural changes needed to improve the health and safety of the working environment. We recognise that persistent advocacy and research to support broader systems change are needed.^[110,111]^ As previously suggested,^[8]^ improved routine reporting of the incidence of TB, COVID-19, and other occupationally-acquired illnesses among HWs will help monitor the longer-term effects of preventive measures and help drive advocacy.

## Conclusion

SARS-CoV-2 and *Mtb* are transmitted via aerosol. HWs are at high risk of infection. The use of surgical masks in frontline settings is inappropriate. Fit-tested particulate FFP respirators provide better protection against infectious aerosols than surgical masks, are already recommended for use by all HWs in high TB burden countries and many COVID-19 pandemic settings, and should be worn routinely to protect HWs against TB and COVID-19.
